# A Single-Center Experience: Integrated Management in Primary Central Nervous System Lymphoma

**DOI:** 10.7759/cureus.75022

**Published:** 2024-12-03

**Authors:** Justin M Kalka, Alexander M Kravets, Bartosz Sokol

**Affiliations:** 1 Surgery, Poznan University of Medical Sciences, Poznan, POL; 2 Neurosurgery, Poznan University of Medical Sciences, Poznan, POL

**Keywords:** awake craniotomy, immunocompetence, neurosurgery, neurosurgical oncology, primary central nervous system lymphoma

## Abstract

The present study reports a single-center experience conducted at Józef Struś Multispecialty City Hospital in Poznań, Poland, in diagnosing and treating two patients with primary central nervous system lymphoma (PCNSL), one immunocompetent and one immunodeficient (AIDS). PCNSL is an extremely rare neoplasm with a poor prognosis and non-specific treatment on the basis of immunocompetency. Standard treatment consists of high-dose methotrexate (HD-MTX) being the background of a multimodal therapy, including other chemotherapeutic agents with and without radiation. To our knowledge, no alteration in management exists in immunocompromised individuals and so patients are subject to standard treatment options. Differences in patient management due to immunocompetency may necessitate separate protocols.

The immunocompetent patient followed a more typical course, while the immunodeficient patient required balancing lymphoma treatment with the risks of opportunistic infections and drug interactions. These cases underscore the importance of tailored therapeutic approaches based on immune competency, aiming to improve outcomes for PCNSL.

## Introduction

Primary central nervous system lymphoma (PCNSL) is a rare extranodal non-Hodgkin's lymphoma (NHL) emerging from the brain, eyes, spine, and cerebrospinal fluid (CSF) with predictably low incidence of metastasis [1]. The majority (>90%) of PCNSL cases are classified as diffuse large B-cell lymphoma (DLBCL) [1,2]and 3% to 4% of CNS tumors are diagnosed as PCNSL [3]. Immunodeficiency is the only known risk factor. Individuals with Human Immunodeficiency Virus (HIV) infection harbor a substantially elevated risk of developing PCNSL compared to the general population [2,3].

Due to localization within the central nervous system (CNS), PCNSL is difficult to diagnose and treat. Diagnosis is typically made with a combination of magnetic resonance imaging (MRI), CSF analysis, and stereotactic biopsy. 

Despite the rarity of this disease, PCNSL is often associated with a poor five-year survival rate primarily ranging from 30-50% with variability predominantly attributed to morphology of the tumor and treatment protocol [1-3]. Immunocompromised patients, including HIV-infected and immunosuppressant patients, have an increased risk for acquiring PCNSL [1,2]. The treatment regimen typically consists of high-dose methotrexate-based chemotherapy and whole-brain radiotherapy. The efficacy of tumor resection is controversial. Although PCNSL chemotherapy leads to improved survival, recurrence rates are significant at two years post-remission [1,4].

Along with prevalence, clinical presentation and treatment are interlinked with patient immunocompetence. Although treatment largely varies on a case-by-case basis, there is still a need for standardized integrated management. Additionally, tumor resection may adequately ameliorate neurological symptoms prior to initiation of chemotherapy. In this report, we present two cases where differences in patient management were observed based on patient immunocompetence.

## Case presentation

We present two cases of PCNSL differing significantly in patient management. The diagnosis was made in each case via immunohistochemistry. Systemic lymphoma was ruled out through whole-body computed tomographic (CT) scan with and without intravenous contrast, and positron emission tomography (PET) scan. Additionally, CSF analysis and cytology presented negative for detection of cancer cells. Currently, both patients are undergoing high-dose methotrexate chemotherapy with plans for consolidation therapy via radiation. 

Case 1

**Figure 1:**
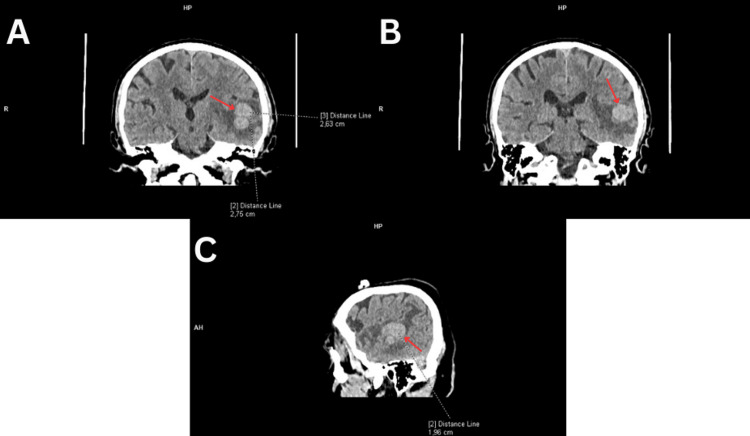
A-C. Head computed tomography without contrast. A faint heterogeneous mass in the left temporal lobe (red arrows) is seen with midline shift.

A 64-year-old male was referred to our department from an outside hospital, for follow-up on an intracerebral mass detected incidentally on CT head scan with contrast. The patient in our department presented with progressive worsening of neurological deficits and an expanding brain mass on non-contrast head CT (Figure 1). On admission he presented with a history of new-onset epileptic seizures and speech impairment with episodic loss of comprehension. 

**Figure 2:**
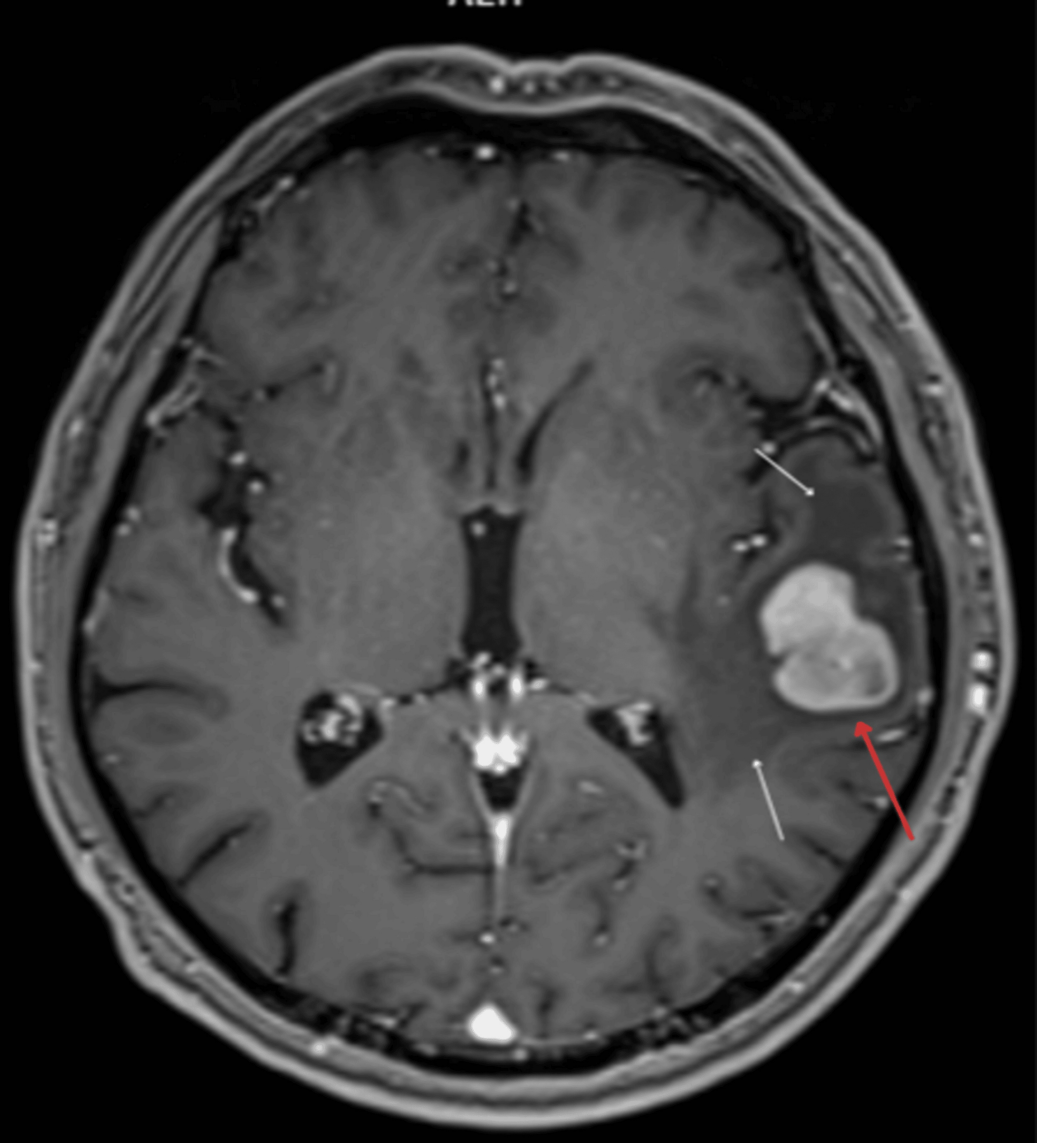
Brain magnetic resonance imaging (MRI). In the left temporal lobe, an irregular hyperdense focal lesion (red arrow) measuring 27x20x25 mm (APxRLxCC) surrounded by a hypodense, finger-shaped zone of edema (white arrows).

Brain MRI on admission showed a heterogeneous lesion in the left temporal lobe measuring 27x20x25 mm with surrounding edema (Figure 2). The edema subtly models a triangle. Thickening of the mucosa of the maxillary, ethmoid and left frontal sinuses are also present. No mass effect was reported.

The care team opted for tumor resection to ameliorate symptoms prior to initiation of chemotherapy. The decision was made to perform an awake craniotomy with intraoperative neurological monitoring to observe the condition of the patient during surgery. The awake craniotomy consisted of speech comprehension monitoring while the patient is conscious, allowing the surgeon to maximize the area of resection while limiting the extent of damage to critical brain function. Psychological assessment occurred during the surgery to monitor temporal lobe function. The temporal lobe was temporarily inhibited using electrical stimulation, helping to delineate the extent of resection required. The patient tolerated the procedure well.

The surgery consisted of a left-sided temporal craniotomy with awakening and neuromonitoring with the goal of removing the tumor mass located in the left temporal region. Local anesthesia with lidocaine and bupivacaine was administered. A Mayfield clamp was applied and the patient was connected to neuromonitoring. The surgical field was cleaned and injected with an additional dose of bupivacaine. A skin and muscle incision was made on the right temporal side. An entrance incision was made through the dura in a cruciform shape, mapping the superior and inferior temporal gyri; avoiding injury to the speech center. A corticectomy was performed in the upper and middle temporal gyri to resect the tumor. Hemostasis of the cavity was achieved. The bone flap was reattached using craniofix. A subcutaneous drain was inserted. Layered suturing of the wound was performed then dressing was applied. The resected mass was sent to pathology. No intraoperative changes in speech comprehension were observed. 

**Figure 3:**
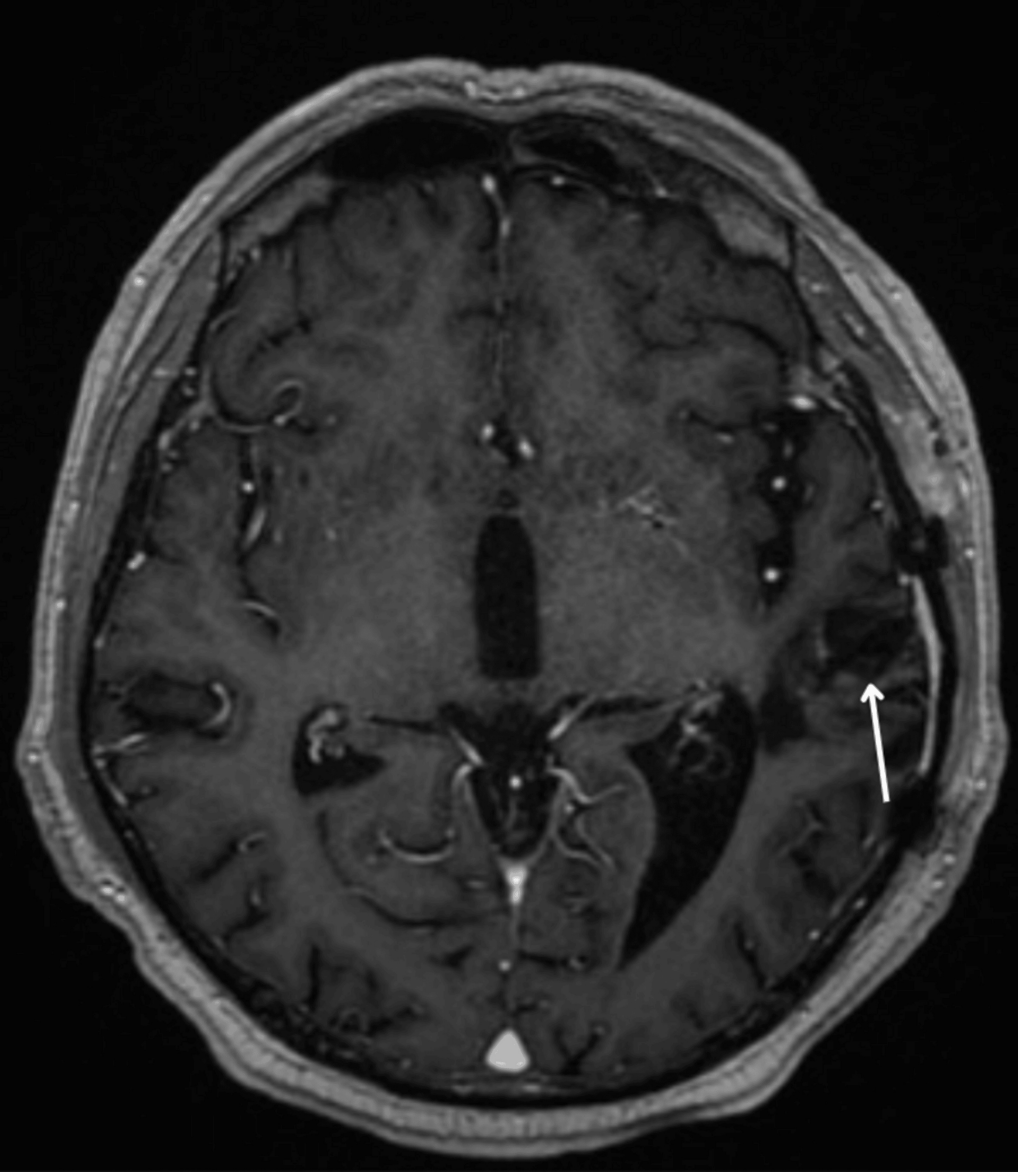
A control brain MRI was performed at three months postoperation. A cavity is shown following resection, edited with slight contrast enhancement for better visualization.

The patient had an uneventful postoperative recovery and was discharged with plans for follow-up in clinic. Serum testing was negative for HIV. A control brain MRI was performed at three months postoperation. A cavity is shown following resection, edited with slight contrast enhancement for better visualization (Figure 3).

On pathologic examination, the mass was diagnosed as primary DLBCL of the CNS. Immunohistochemistry demonstrated positive staining for CD-20, Ki-67 (90%), and leukocyte common antigen (LCA).

No significant changes in motor activity and speech production were observed on follow-up. The patient tolerated a combination chemotherapy regimen of high-dose methotrexate, rituximab, and berubicin without any complication or reported issues. 

Case 2

A 37-year-old male AIDS patient was referred to our department for neurologic changes and a brain lesion found on non-contrast head CT. The patient presented to our department with slight psychomotor retardation and normal gait.

The patient also had multiple opportunistic infections including cytomegalovirus (CMV)-retinitis, *Pneumocystis jirovecii* pneumonia, aspergillus infection, and CMV colitis. Lymphoma was on the differential along with toxoplasma and tuberculoma. The patient tested negative for serum IgG anti-toxoplasma antibody and John Cunningham (JC) virus. 

**Figure 4:**
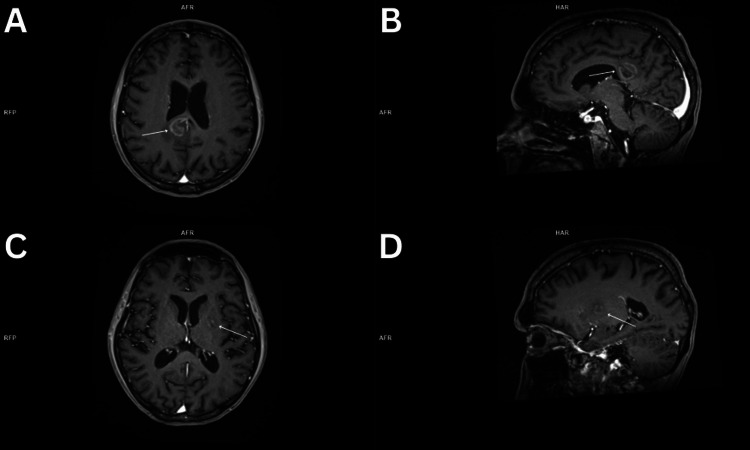
A-D. Brain MRI showing two lesions. (A,B) The larger mass is an expanding heterogeneous lesion in the right frontal lobe measuring 22x23x19 mm surrounded by a small zone of edema.

Brain MRI showed two lesions, one of which is an expanding heterogeneous lesion in the right frontal lobe measuring 22x23x19 mm surrounded by a small zone of edema (Figure 4). The tumor was observed to shape the posterior horn of the right corpus callosum. 

Due to risks pertaining to the location of the tumor, surgical resection was contraindicated per care team. The patient agreed to undergo a stereotactic biopsy. A small sample was obtained without issue and sent to pathology.

The lesion was diagnosed as primary DLBCL on pathologic examination. On immunohistochemistry, the specimen stained positive for leukocyte common antigen, CD20, and Ki67 (70%).

## Discussion

Diagnosis

The diagnosis of PCNSL can be particularly challenging due to patients presenting with nonspecific symptoms, which often mimic those of other neurologic conditions. This complexity is compounded in immunocompromised patients. Early and accurate diagnosis is crucial for effective treatment. Patients delayed in starting chemotherapy would have benefited from a prompt biopsy.

In our second case, the patient's HIV status led to a quicker diagnostic process. Immunodeficient patients are more likely to undergo routine surveillance for opportunistic infections which lead to earlier tumor mass detection.

PCNSL is frequently misdiagnosed as it shares symptoms with and/or mimics other diseases [5,6]. Consistent with existing literature, our case illustrates the diagnostic challenges in differentiating PCNSL from other diseases in immunocompromised patients. The difficulty in distinguishing between lymphoma and other diseases often delays definitive treatment, emphasizing the need for heightened clinical awareness and improved diagnostic protocols.

Surgery

Surgical resection in patients with PCNSL is a topic of ongoing debate. Newer studies show that surgical resection, in conjunction with chemotherapy, may correlate with increased one-, three-, and five-year overall survival (OS) [2,7]. However, further research is required to solidify these benefits in common practice and to determine the most effective treatment protocols for PCNSL. An increase in well-documented PCNSL cases may provide ample evidence for surgical benefits in common practice without controversy.

Our case adds to the emerging view that surgery, despite its risks, can be beneficial in the integrated management of PCNSL. This aligns with recent studies indicating that surgical resection, alongside chemotherapy, improves OS, particularly in patients who can tolerate aggressive treatments [2,7,8]. However, despite positive results in limited modern research every case is unique and may not permit surgery. In our immunocompromised patient surgical resection was not a viable option due to the location of the tumor defining the complexity of routine surgery.

The literature presents mixed opinions on the role of surgery in PCNSL management. Some studies argue against surgical intervention due to the infiltrative nature of the tumor and the potential for significant complications. Nevertheless, our findings contribute to the growing body of data on surgical cases in conjunction with chemotherapy, whether or not surgical resection may offer a survival advantage. 

Case comparison

The immunocompetent elderly patient presented with a more typical PCNSL progression, while the younger immunocompromised patient had a more complex clinical picture due to concurrent AIDS-related infections. This suggests that immunocompetency not only affects the timing of diagnosis but also the range of differential diagnoses considered.

Due to regular medical surveillance for opportunistic infections, PCNSL may be discovered earlier in the immunocompromised population. However, immunodeficiency can also complicate the clinical picture, making it difficult to isolate PCNSL from existing conditions and potential complications. Our findings reinforce these observations, demonstrating the diagnostic complexities and the need for a nuanced approach in managing PCNSL in immunocompromised patients.

In the immunocompetent patient, treatment regimen included a combination chemotherapy regimen of high-dose methotrexate and rituximab. High-dose methotrexate is generally accepted as first-line treatment in PCNSL management [1,4,7,9]. However, conflicting studies discuss rituximab efficacy. In one study, high-dose methotrexate in conjunction with rituximab improves patient outcomes [1], whereas another contradicts this claim and states that rituximab is commonly used for systemic DLBCL with limited reported efficacy in PCNSL, but is still used due to the relatively low toxicity [9]. The problem then lies with error in a definitive treatment plan for PCNSL cases.

The first step in recovery is seeking treatment and receiving a definitive diagnosis. Among our two cases, the diagnosis was improperly handled leading to a delayed treatment plan. 

In the first case, the patient was incorrectly treated as an oncological patient instead of a neurosurgical patient. Typically, in oncological patients tumors are slow growing and take longer to treat. When dealing with a PCNSL patient aggressive treatment is beneficial in patients who can tolerate it [7]. OS in PCNSL left untreated is estimated at three to six months and five-year OS with treatment remains 20-25% [8]. Although a brain lesion was discovered early on PCNSL wasn’t properly discussed with the patient thus a stereotactic biopsy was not performed. Stereotactic biopsy is not routine for PCNSL in some hospitals, this may cause issues in patient communication and urgency in treatment. 

As for the immunocompromised patient, treatment was also delayed despite an earlier differential which included PCNSL. Imaging revealed a brain lesion believed to be either PCNSL, toxoplasmosis or progressive multifocal leukoencephalopathy (Figure 4) [10]. PCNSL was not only put on the differential, but the other suspicions, toxoplasmosis and JC virus gave negative serum results. These negative results should indicate a biopsy of the visualized lesion to confirm a diagnosis. This unorganized process advocates for a need in a clinical standard. A stereotactic biopsy procedure in patients with brain lesions who have tested negative for toxoplasmosis and JC virus with a differential which includes PCNSL should be routinely recommended. The location of the tumor also presented an issue with location near the lateral ventricle, contraindicating surgical resection. It should also be stated that patients with brain lesions in difficult locations may still go for stereotactic biopsy, as opposed to surgical resection, to reach a definitive diagnosis.

Diagnosis is the most important step in PCNSL and should be structured to increase survival rates. Not only was there a lack of urgency in the presented cases for PCNSL, but the condition lacked a mainstream process to confirm it. Stereotactic biopsy must be included in standard workup when presented with sufficient evidence.

## Conclusions

One of the major discussions brought up in this paper is the efficacy of surgery in patients with PCNSL. The approach novel research should approach is class of surgery in PCNSL. We believe in separating surgical resection into classes including multifocal, unifocal, partial resection, and complete resection. If the tumor is unable to be fully resected due to close proximity of essential brain structures, partial resection may be opted for given that the literature advocates it. This new classification system, if implemented, may reveal new trends in data that have not been properly discussed before, such as how separate chemotherapy regimens work better with or without complete surgical resection.

We also propose that future studies dealing with a greater number of individuals should include immunocompetency on long-term patient survival. There is a gap in the current common literature for including immunocompetency when taking into account OS. Immunocompetency should be just as crucial a factor as age, race, or gender in PCNSL epidemiological studies as it may provide a specialized treatment protocol in immunodeficient individuals instead of a generalized standard regimen. This approach can be met by defining immunodeficiency with low white blood cell (WBC) count. Including this as an additional criterion in future papers may provide correlations in PCNSL that reveal efficacy of structured, but also specialized patient-based treatment in different regimens.
